# Physiological, perceptual, and technical responses to continuous and intermittent small-sided games in lacrosse players

**DOI:** 10.1371/journal.pone.0203832

**Published:** 2018-10-03

**Authors:** Richard Hauer, Antonio Tessitore, Nicole Binder, Harald Tschan

**Affiliations:** 1 Centre for Sport Science and University Sports, University of Vienna, Vienna, Austria; 2 Department of Movement, Human and Health Sciences, University of Rome “Foro Italico”, Rome, Italy; Universita degli Studi di Verona, ITALY

## Abstract

**Purpose:**

The present study was designed to investigate the influence of two distinct small-sided game (SSG) regimes on physiological, perceptual, and technical parameters in male elite lacrosse players.

**Method:**

Data were collected in twelve elite male Austrian lacrosse players (25.8 ± 5.5 years; 80.1 ± 7.7 kg; 178.5 ± 6.2 cm). Players’ were assigned to an intermittent (SSG-I) or a continuous (SSG-C) SSG regime, respectively. Regimes were equated for total practice time, but not active playing time. SSG data from eight sessions of 3 vs. 3 self-regulated match-play were collected along a 4-week pre-season period. Players’ YoYo-Level 1 (YYL1) performance before and after the training intervention was recorded. Further, heart-rate (HR), rating of perceived exertion (RPE), physical activity enjoyment scale (PACES), and technical actions during and after SSG sessions were analyzed.

**Results:**

Both SSG regimes showed improvement with medium to very large effect sizes (ES) in YYL1 total distance covered pre- to post-intervention (SSG-C mean-difference ± SD: 840 ± 299 m; p = 0.003; d = 1.08; CI = 0.60 to 1.56 and SSG-I: 607 ± 274 m; p = 0.003; d = 1.25; CI = 0.66 to 1.85 respectively). Higher %HR_max_ values with very large ES (92 ± 0.6%; p = 0.002; d = 5.33; CI = 2.78 to 7.88) and time spent in HR zone 4 (1248.0 ± 122.7 s; p = 0.000; d = 3.43; CI = 2.31 to 4.55) were observed for SSG-C. No differences between regimes were found for any of the assessed technical actions, global RPE, and PACES scores.

**Conclusions:**

Both SSG regimes investigated in this study were effective in improving YYL1 performance. Further, findings indicate that the regime does not influence players’ subjective feelings and technical actions in SSG play. However, SSG-I in lacrosse specific training could have additional benefits such as lower signs of fatigue. Further, breaks can be used to give technical and tactical inputs by coaches.

## Introduction

Lacrosse is a Native American stick and ball invasion game [1]. Today, according to international rules it is played with 10 players per side on a 110m x 60m pitch, in 20min quarters, and unlimited interchange. The game is characterized by intermittent high-intensity activity, collisions, and rapid changes of directions [2]. With time, it has become one of the fastest growing team sports in the United States of America. Increased popularity is also noticeable in other areas of the world [3–6]. Such growth supports the need for more research into the sport. However, despite the importance that specific knowledge of the physiological and game demands could have on further improvements of lacrosse performance, most of the recent research has focused on issues related to injuries [7–10]. In contrast, few studies have investigated the physiological demands or technical and tactical needs of lacrosse play during match-play and training [2, 3, 11–18]. Indeed, even if similarities with other field games do exist [11], training and competition demands have often been adopted from these sports, rather than being based on the specific characteristics of lacrosse. Consequently, the nature and specificity of lacrosse demands still presents several areas requiring further investigation. Furthermore, much less is known about training loads and the implementation of particular forms of training.

Compared to regular match-play, small-sided games (SSG) are played with reduced number of players and/or pitch size [19], as well as modified rules [20]. For this adaptability, SSGs are often used by coaches of different field games to enhance technical and tactical skills, and aerobic fitness of their players [21]. To support this choice, it is also well established that SSGs can improve game specific endurance capacity [22, 23], develop technical and tactical abilities in game-specific conditions [24], and provide a positive transfer to match-play [25]. Previous studies in soccer [23, 26], rugby [27–29], and basketball [30, 31] give evidence that SSGs are equally effective as traditional aerobic conditioning programs at improving aerobic capacity. Further, SSGs evoke a high heart rate (HR) response similar to a short-duration intermittent running activity. They are performed with an intensity above 90% of HR_max_, which has been reported as being effective to enhance aerobic capacity [26, 32–34]. In addition to the positive effects on aerobic capacity, general research has also demonstrated how SSGs can combine motor learning and team cohesion components. Furthermore, they can be considered a more enjoyable form of training compared to traditional aerobic conditioning [23, 28].

It is well known that SSG training intensity is affected by numerous variables, such as pitch size and number of players [35], rule modifications [30], goalkeeper presence [36], and coach encouragement [37]. However, the considerable body of literature on this issue has also produced some contrasting results over a variety of sports. For instance, analyses of HR response to different pitch sizes. Studies by Kennett et al. [29], and Alti et al. [38] found a related increase of HR_max_, as well as lactate concentration and rating of perceived exertion (RPE), while Kelly and Drust [39] reported lower HR values with increased pitch size. Nevertheless, Halouani et al. [28] suggest that larger pitch dimensions can be used to maintain high intensity throughout exercise. Findings by Brock, Christopher [40] support this hypothesis and additionally observed differences of technical skill indicators of Australian football players during SSGs. Similarly, Martone, Giacobbe [41] reported higher exercise intensity and changes in technical actions for larger areas per player in young soccer players. Moreover, some studies [27, 35] reported that a reduced number of players was closely linked to an increase in HR and RPE responses. In the same manner, rule changes have shown effects on technical and tactical parameters [28]. Indeed, mixed offensive and defensive situations, the “one-touch” and “man-marking” rules showed to elicit higher SSG training intensity in soccer [42]. Investigations by Atli et al. [38], and Rampinini et al. [37] highlighted the importance of coach encouragement to achieve a high training intensity during SSGs. Their findings resulted in higher HR, lactate and RPE responses stimulated by active and consistent coach encouragement. Fanchini et al. [43] found a difference between intensity and SSG duration. Results showed highest HR in 4-minute bouts compared to 2- and 6-minute SSGs. For technical actions no differences were found.

Another factor affecting the SSG performance is the training regime. Recent SSG studies focused on traditional interval training regimes based on work intensity and duration, recovery type and duration, and total work duration. In contrast, only a small number of studies dealt with a continuous SSG regime that used longer duration without recovery in between [28]. In this regard, Hill-Haas et al. [44] found significantly higher RPE scores and HR_max_ values during continuous SSG regime compared to interval SSG. These findings are supported by Koklu, Alemdaroglu [45] who reported higher intensities during continuous SSGs compared to interval formats. Another study by Koklu [36] investigated the effects of interval and continuous SSG regimes with opponent groups composed by three different sets of player numbers. Results showed similar physiological response regardless the training regime. Similarly, Christopher, Beato and Hulton [46] found no physiological or subjective rating differences, and only minor technical manipulations between continuous and intermittent conditions. It appears that both intermittent and continuous training regimes can be used for physiological adaptions and match-specific conditioning. The effects on technical actions still seems to be unclear with contrary results in recent research.

Taking into account the above mentioned considerations, it has been shown that SSGs can be an effective tool to develop match-specific aerobic capacity. However, considering the wide variety of variables that influence technical, tactical and physiological parameters, there is some uncertainty about the most appropriate SSG regimes. In this sense, there is still a lack of research exploring the effect of continuous vs. interval SSG training regimes in different team sports. Further, no research about the organization and implementation of SSGs in the sport of Lacrosse have been done so far. In the same manner, no data about the physiological or technical parameters in lacrosse specific SSG play exists. Therefore, the purpose of this study was to explore the influence of continuous vs. interval SSG training regimes on physiological, perceptual, and technical responses of lacrosse players during SSGs. Results of this study will allow a better understanding of the role of the training regime and provide new insights into SSG design for coaches, practitioners and researchers. It was hypothesized that both training regimes would improve the athletes’ aerobic capacity. On the other hand, mean HR_max_ and time spent in different HR_max_ zones during training sessions were assumed to differ. Similarly, differences for subjective feeling, evaluated through RPE and Physical Activity Enjoyment Scale (PACES) score, were expected. Further, it was hypothesized that the training regime would influence some technical parameters such as possession time, complete and incomplete passes, number of shots, and groundballs.

## Material and methods

### Experimental approach to the problem

To test the hypothesis, players were allocated to either a continuous (SSG-C) or intermittent (SSG-I) SSG training regime, depending on their YYL1 performance and technical skill level. Both SSG regime training sessions lasted for 25 minutes’ played 3 vs. 3 self-regulated match-play on an indoor basketball court twice per week. %HR_max_, time spent in intensity zones, technical actions, and RPE were collected for each training session. Additionally, at the end of the 4-week intervention the player’s total enjoyment for SSGs training was recorded using the PACES questionnaire.

### Participants

Twelve male lacrosse players (age 25.8 ± 5.5 years; body mass 80.1 ± 7.7 kg; height 178.5 ± 6.2 cm) participated in the study. All participants were members of the same club team and the Austrian national team practice-squad, which was in preparation for the European Box Lacrosse Championships. Additional to intervention, participants undertook a standardized strength training, twice a week and were asked to follow their normal dietary guidelines. The study was approved by the University of Vienna Ethics Review Board (Reference Number: 00241) and was conducted in accordance with the Declaration of Helsinki. All subjects provided informed consent after reading a description of all research procedures and received guarantee on data anonymization.

### Procedure

The SSGs were performed along a 4-week pre-season period, twice a week, for a total of eight training sessions, during which players’ were divided in four squads. The selection of each squad was made by the coach, considering the players’ individual skill and fitness levels, to avoid mismatches and imbalance between opponent squads. For the same reason, the players’ participation within the same squad and the opposition of the same two squads was maintained constant for all the experimental period. Thus, over the 4-week period, two squads were always submitted to an intermittent SSG regime, while the other two performed a continuous one. Each pair of squads was familiarized according to the allocated SSG regime. All SSGs were played self-regulated according to free competitive match-play rules without any instructions or coach encouragement. The objective of the SSG was to keep ball possession and hit one of the two corner targets of the opponent’s goal (1.22 x 1.22 m) placed at the end line of the court. After a goal was scored the ball started in the goal-crease area of the team that had received the goal. To keep the play fluent and intensity high a ball was immediately replaced when ever hit out of play. All SSG sessions were completed after a standardized 20 min warm-up of:

- 4 minutes of lower body tissue quality using foam roller
- 4 minutes of lying, sitting and standing, activation exercises with 10 repetitions each
- 2 minutes of correction exercises for ankle and hips
- 10 minutes of thermogenic and dynamic warm-up exercises, including functional movements and sprints

All SSG sessions were played at an indoor basketball court sized 28 x 15 m, and a 3 vs. 3 playing format, resulting in a mean area per player of 70 m^2^. The intermittent regime (SSG-I) was implemented through 4 bouts of 4-minute work interspersed by 3-minute of active recovery. During the recovery phase players were encouraged to keep the intensity in a range of 65–75%HR_max_ while passing a ball in pairs. The continuous regime (SSG-C) comprised 25 minutes of play without rest intervals. Same total session time of 25 minutes for both regimes, with different active playing time (SSG-I: 16 and SSG-C: 25 min) was chosen to evaluate differences in physiological outcomes of different high intensity times. Further, the technical aspect of passing during the active recovery phase in SSG-I (9 min), was seen as a training content for skill improvement and has been a part of the SSG-I training session. Therefore, an equal training time for both regimes of 25 minutes was taken into account for the process of analysis.

Before the commencement and at the completion of the 4-week SSG period, a YoYo Intermittent Recovery Level 1 test (YYL1), which is a valid and reliable test used in different team sports [47–50] was completed to determine players’ HR_max_ and provide an indication of aerobic fitness status. The players were then ranked according to distance travelled during the YYL1 test, assessed at the beginning of the intervention (Table 1), and their lacrosse skill level score rated by their coaches own appraisal using a 5-point likert-scale (from 1 = “below average” to 5 = “outstanding”). Depending on the YYL1 rank and the skill rate, players were allocated to teams with even strength.

**Table 1:** Anthropometric, physiological, and perceptual parameters. Results are presented as mean ± SD (95% CI) for continuous and intermittent SSG regimes.

SSG—Parameters	N	SSG—Intermittent	N	SSG—Continuous	Significance level (p)	Effect size (d)	95% CI
**Anthropometric parameters**							
Age (years)	6	24.9 ± 7.3	6	26.7 ± 3.5			
Height (cm)	6	177.9 ± 8.7	6	179.1 ± 2.7			
Body mass (kg)	6	81.2 ± 9.4	6	79.0 ± 6.3			
BMI	6	25.6 ± 1.7	6	24.6 ± 1.8			
**Physiological or perceptual parameters**							
YYL-1 pre-intervention (m)	6	1793 ± 407	6	1460 ± 588	0.265	0.60	-0.55 to 1.74
YYL-1 post-intervention (m)	6	2400 ± 462	5	2312 ± 350	0.735	0.18	-0.98 to 1.34
YYL-1 differences between regimes (m) (inter-difference)	6	-233 ±173	5	+233 ±173	0.210	-0.67	-1.83 to 0.49
YYL-1 improvement for regime (m) (intra-difference)	6	607 ± 274**	5	840 ± 299**	0.003* / 0.003*	1.25 / 1.08	0.66 to 1.85 / 0.60 to 1.56
Mean HR_max_ (%) total session	6	86 ± 3*	6	92 ± 1*	0.002*	5.33	2.78 to 7.88
Mean HR_max_ (%) work bouts	6	90 ± 2	6	92 ± 1	0.08	1.85	-0.41 to 4.11
Mean RPE rank	6	6.1 (37.0)	6	6.8 (41.0)	0.748	0.09 (r)	
Mean PACES score	6	96.0 ± 16.4	6	90.5 ± 15.9	0.568	0.29	-0.81 to 1.39

Intra- and inter-differences for regimes

*Significance (p<0.05)

**Significance (p<0.001)

Heart rate was measured during all SSGs sessions by means of a belt heart rate monitor positioned on the players chest (Polar Team^2^ System, Polar Electro, Finland). Maximum HR (HR_max_) obtained from the YYL1 test was used to determine percentage of HR_max_ (%HR_max_) during the SSGs for each player. Mean %HR_max_ for each player during all SSGs were calculated. Further, four heart rate zones were classified on the basis of %HR_max_ and the time spent in intensity zones: zone 1 (<75%), zone 2 (75–85%), zone 3 (>85–90%), and zone 4 (>90%) was reported. Then, players’ RPE was obtained 15 minutes after the end of each SSG session using the 6–20 Borg’s scale [51]. Finally, according to each regime, to assess the technical actions SSGs were recorded using a video camera (Coolpix P610, Nikon, Japan), which was placed at the stands of the gym ensuring that the whole court was visible at all times. A data collection system for technical analysis was designed with Microsoft Excel 2010 containing the following parameters: day, regime, period, team, number of possession, duration of possession, number of incomplete passes, number of complete passes, number of shots on goal, number of shots missed, number of goals, and number of groundballs. The actions during the active recovery phases for SSG-I were excluded, to analyze only the technical parameters during active game-play time. All data were collected by two researches with at least 10 years of lacrosse experience. The intra observer reliability of the data collection system was tested with collected data from two randomly chosen training sessions, one of each regime. The observers analyzed the training sessions twice with seven days in between first and second time. To assess intra-observer agreement and reliability kappa statistic equation was calculated [52, 53]. The 581 events recorded by the observers for the first time were used as a total of recorded events. Further percentage error for events was evaluated [53, 54]. The evaluated numbers leaded to, p_o_ = (581–7)/581 = 0.988 and p_c_ = 1.21/581 = 0.002 and a kappa of 0.988. The same equations were used to calculate Cohen’s kappa for possessions (k = 0.934). With all kappa values of at least 0.934 or higher intra-observer reliability is considered proven.[52]

In addition, the player’s total enjoyment during the entire SSGs training intervention was recorded using the PACES questionnaire, which is proven to be a valid and reliable tool to assess physical activity enjoyment [55, 56].

### Statistical analyses

All statistical analyses were performed with the IBM software SPSS Version 22 (SPSS, Chicago, IL, USA). Assumptions of normality were verified by the Shapiro-Wilk test and histograms. To determine effects of SSG regime (intermittent or continuous) an independent t-test was used on the dependent variables %HR_max_, PACES score, and all technical parameters. For RPE values a Mann Whitney test was performed. A one-way ANOVA was used to analyze SSG regime (intermittent or continuous) differences on the time spent in the different intensity zones (1–4). A repeated measurement ANOVA was performed to understand the effect of SSG regime (intermittent or continuous) on YYL1 test results pre- and post-intervention. The data are reported as mean ± SD unless stated otherwise. For comparisons between groups and to provide an estimate of effects Cohen’s ES was calculated using spreadsheets by Hopkins [57]. The magnitude of the inferences was determined as Small (d = 0.2–0.5), Medium (d> 0.5–0.8), Large (d> 0.8–1.3), and Very large (d> 1.3) [58, 59]. The significance level for all tests was set at p ≤ 0.05. All calculations are based on a 95% confidence interval (CI) calculated using spreadsheets by Hopkins [57].

## Results

### Physiological analysis

Pre- and post-intervention YYL1 test results are presented in Table 1. No difference between regimes was reported (SSG-I: 1793 ± 408 m and SSG-C: 1460 ± 558; p = 0.265; d = 0.60; CI = -0.55 to 1.74), pre-intervention. Both training regimes showed an improvement with an ES ≥ medium (SSG-C mean difference: 840 ± 299 m; p = 0.003; d = 1.08; CI = 0.60 to 1.56 and SSG-I mean difference: 607 ± 274 m; p = 0.003; d = 1.25; CI = 0.66 to 1.85) in total distance covered post minus pre intervention. There was no difference between regimes in improvement over time (F = 1.82; p = 0.210; d = -0.67; CI = -1.83 to 0.49). Similar results were found using the starting YYL1 score as a co-variate (d = 0.70; CI = -1.06 to 2.46).

**Fig 1:**
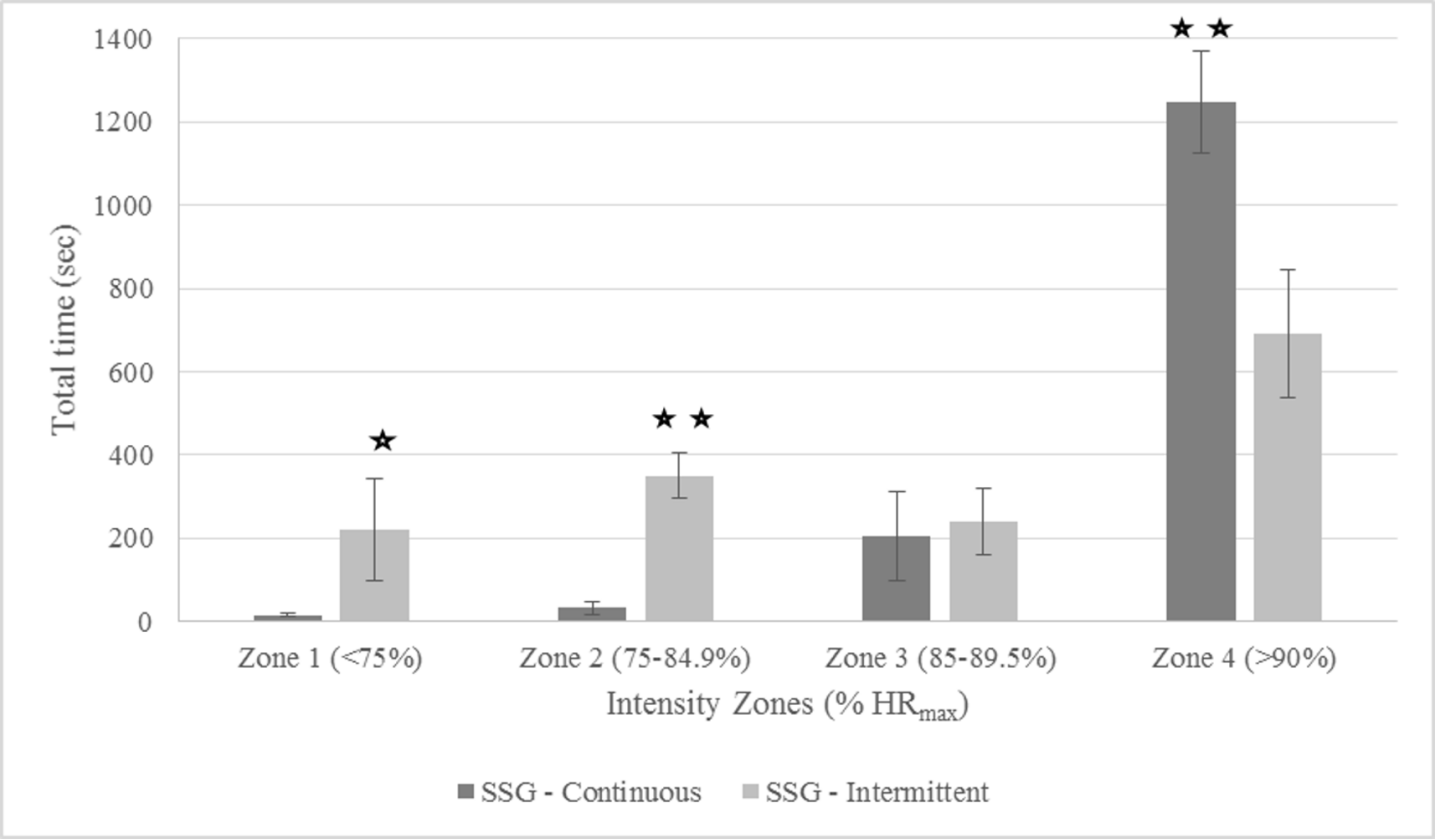
Differences in time spent (sec) in different intensity zones (%HR_max_) during entire intermittent (SSG-I) and continuous (SSG-C) small-sided game sessions. Differences between regimes:*Significance (p<0.05);**Significance (p<0.001).

The acute physiological responses in a training session expressed through mean %HR_max_ showed higher values with a very large ES for SSG-C compared to SSG-I (SSG-C: 92 ± 0.6% and SSG-I: 86 ± 2.8%; p = 0.002; d = 5.33; CI = 2.78 to 7.88). Taking into account only the work bouts excluding breaks for the SSG-I no differences in mean %HR_max_ were found (SSG-I: 90 ± 2.4%; p = 0.082; d = 1.85; CI = -0.41 to 4.11). With regard to relative time spent in each intensity zone, for the whole training session (including active recovery time for SSG-I), differences with very large ES were found between regimes as shown in “Fig 1”. SSG-C spent more time in zone 4 (SSG-I: 691.93 ± 154.18 s and SSG-C: 1248.0 ± 122.7; p = 0.000; d = -3.43; CI = -4.55 to -2.31) compared to SSG-I with more time in zone 1 (SSG-I zone 1: 221.8 ± 123.5 and SSG-C: 15.17 ± 3.79 s; p = 0.002; d = 23.63; CI = 8.80 to 38.47) and zone 2 (SSG-I: 350.5 ± 53.5 and SSG-C: 32.75 ± 14.31 s; p = 0.000; d = 11.85; CI = 9.68 to 14.02). No differences were found for zone 3 (SSG-I 241.4 ± 80.6 m and SSG-C: 205.9 ± 108.3; p = 0.534; d = 0.32; CI = -0.81 to 1.46).

Similarly results have been found, for the percentage of relative time spent in each intensity zone, regarding only active playing time (SSG-C: 25min. and SSG-I: 16min., respectively). SSG-I showed higher values and very large ES in zone 1 (SSG-I: 4.6 ± 2.0 and SSG-C: 1.0 ± 0.0%; p = 0.007; d = 6.69; CI = 2.74 to 10.64) and zone 2 (SSG-I: 12.4 ± 5.0 and SSG-C: 2.17 ± 1.00%; p = 0.004; d = 5.36; CI = 2.56 to 8.16). Conversely, SSG-C showed higher values and an ES ≥ small in zone 4 (SSG-I: 65.3 ± 14.1 and SSG-C: 83.3 ± 8.1%; p = 0.021; d = -1.46; CI = -2.71 to -0.21). No differences were found for zone 3 (SSG-I: 18.4 ± 8.4 and SSG-C: 13.5 ± 7.3%; p = 0.330; d = 0.48; CI = -0.63 to 1.59).

### Perceptual characteristics

No differences and relevant ES in global RPE were found between training regimes (SSG-C mean rank: 6.8 and SSG-I mean rank: 6.2; p = 0.748; r = 0.09). Similarly, there were no differences in PACES scores (SSG-I: 96.0 ± 16.4 and SSG-C: 90.5 ± 15.9; p = 0.568; d = 0.29; CI = -0.81 to 1.39), as presented in Table 1.

### Technical actions

Technical actions (Table 2) were only assessed for active game-play time. No differences were found for any of the assessed technical parameters per possession. Similarly, there were no differences for any technical parameters over time between regimes.

**Table 2:** Technical-Parameters per possession. Results are presented as mean ± SD (95% CI) for continuous and intermittent SSG regimes.

Technical–Parameters per possession	SSG—Intermittent	SSG—Continuous	Effect size (d)	95% CI
Events	4.09 ± 3.44	4.24 ± 3.07	-0.05	-0.22 to 0.13
Duration	18.50 ± 15.15	16.83 ± 12.96	0.12	-0.06 to 0.30
Passes incomplete	0.78 ± 1.09	0.99 ± 1.04	-0.20	-0.37 to -0.02
Passes complete	1.03 ± 1.23	0.93 ± 1.08	0.09	-0.09 to 0.26
Shots on goal	0.37 ± 0.51	0.32 ± 0.49	0.09	-0.09 to 0.26
Shots wide	0.39 ± 0.65	0.40 ± 0.61	0.00	-0.18 to 0.17
Goals	0.05 ± 0.22	0.04 ± 0.20	0.05	-0.13 to 0.22
Groundballs	1.46 ± 1.41	1.52 ± 1.33	-0.04	-0.22 to 0.13

## Discussion

SSGs are a frequently used method to elicit a sport-specific aerobic training effect in team sports [28]. However, in current literature there is a lack of evidence to help coaches design sport-specific SSGs in training sessions. Therefore, the aim of this study was to explore the influence of two different SSG regimes (intermittent and continuous) on physiological, perceptual, and technical responses of lacrosse players during SSGs. To our knowledge, the current study is the first to compare the output of different SSG regimes on the performance of lacrosse players, which will improve the awareness on how SSGs can be used for match-specific conditioning and lacrosse specific skill development within training context.

Both SSG regimes investigated in this study were associated with an improvement in the total distance covered during the YYL1 test post-intervention. This improvement in both regimes results indicate SSGs to be a beneficial tool to improve endurance performance of the elite male lacrosse players recruited in our study. The higher values and very large ES for mean %HR_max_ observed during the entire training sessions with SSG-C regime are in line with those registered in a previous study of Hill-Haas et al. [44]. In like manner, the analysis of the time spent in different intensity zones also showed differences between the two regimes. Findings revealed a longer duration spent in high intensity zone 4 for SSG-C. One of the possible reasons for this result is the different work-to-rest ratio between the two regimes. Therefore, to quantify the influence of this ratio on mean %HR_max_, rest periods for SSG-I were excluded to give information about bouts intensity only. It is interesting to note that, contrary to what was expected for mean %HR_max_ values, according the very large ES, %HR_max_ still seemed to be lower during SSG-I. However, results should be interpreted with caution as a CI range of -0.41 to 4.11 shows that more data is needed to underline the findings in this study. Nevertheless, both SSG regimes submitted in this study showed mean HRs above 90%HR_max_. Values > 90%HR_max_ are considered to be ideally suited to improve aerobic fitness [34], supporting the findings of the improved YYL1 test results found in this study. In the same manner, duration spent in intensity zones was relativized by calculating the percentage of time within each HRzone, taking into account only the active playing time (SSG-C: 25min, and SSG-I: 16min). Similar to total HRzone duration values, findings showed higher duration spent in zone 1 and 2, but lower duration in zone 4 for SSG-I compared to SSG-C. Zone 3 showed no difference between the two regimes. These results for %HR_max_ and HRzone suggest that, independent from active playing time, intensity seems to be higher if no breaks are included in SSG regimes. One explanation can be that the ongoing heart rate recovery during a break influences the intensity values at the beginning of the next bout.

In contrast to findings by Hill-Haas et al. [44], in our study no differences between SSG regimes were found for RPE values. Similarly, there were no differences for PACES scores. However, these findings may be somewhat limited due to the study design. In fact, since the main purpose of this study was to investigate the physiological effects of different SSG regimes, our design focused on parallel rather than crossover, randomized trials.

Results for technical actions showed no differences between regimes. Therefore, it is still questionable if an increased fatigue caused by the continuous high intensity exposure during the SSG-C regime influence technical actions during SSG sessions. Even with no evidence in this study the authors think that the adoption of breaks (as used for SSG-I) to perform this specific lacrosse training can stimulate a better quality of SSGs play and improve the response of some technical actions. However, further investigation is needed to prove this hypothesis.

Recent research has shown that SSGs are a common tool used by coaches to improve endurance and develop technical and tactical skills in team sports. Moreover, several studies identified the effects on intensity and technical actions while manipulating different variables such as pitch size [19], number of players, and rule changes just to name a few [28]. The present study provides additional information about the effects that different SSG regimes can have on physiological, perceptual, and technical parameters. In summary, both regimes showed effectiveness to improve the players’ YYL1 performance. In accordance with recent literature on SSGs, the use of a continuous training regime produces higher mean %HR_max_ values, which implies higher exercise intensity and training load. However, contrary to expectations and published findings, our study did not find any differences in subjective player feelings between different SSG regimes. Furthermore, the analysis of technical actions showed no differences between the training regimes.

The results of this study demonstrate that both intermittent and continuous SSG training regimes could potentially be used to improve endurance and work on technical skill development in lacrosse players. On one hand, the fact that the SSG-C regime produced higher exercise intensities, and consequently higher training loads, should be considered when tailoring specific programs for lacrosse players. On the other hand, it still seems to be unclear if technical skill quality during training sessions is affected by the regime. Additionally, a benefit of SSG-I regime is that coaches can use the within-drill rest periods to deliver tactical inputs and implement technical skills to work on players’ skill transfer and decision making abilities. Furthermore, these breaks of exercise continuity during SSGs can be used to modify variables (i.e. technical and tactical) and therefore change effects and outputs within the same training session. However, further research should be undertaken for a better understanding of the impact that SSGs can have on other fitness parameters such as time spent in different speed zones, and number of sprints, performed by lacrosse players. Future investigations should take into account that parallel rather than crossover, randomized trials have been used in this study. Further, the differences in active playing time between regimes could have influenced the outcomes of the present study and not the regime by itself. Beside the aforementioned limitations which have characterized the present study, other interventions like coach encouragement, instructions, and rules changes just to name a few, have not been investigated in the present study. Future research should investigate the aspects to optimize the practical implications.

## Conclusion

In conclusion, the present study has shown that both SSG-C and SSG-I could be used to improve the YYL1 performance of lacrosse players. Findings indicate that exercise intensity and therefore training load are higher summiting a SSG-C regime. This finding represents an important issue that should be taken into account by coaches and trainers for the implementation of SSGs with a goal of lacrosse specific training. In addition, no differences between regimes have been investigated for players’ subjective feelings and assessed technical actions. Thus, given the small differences registered for physiological, perceptual, and technical parameters between SSG-C and SSG-I regimes the authors recommend to choose the intermittent regime for several reasons. Firstly, intensity and training load seem to be lower, while improvement in endurance is almost equal. Secondly, breaks can be used for technical and tactical inputs, and modification of variables to change outputs during the training session. Finally, these benefits of SSG-I could be used to ensure the optimal training outcome and could lead to a higher match play transfer for lacrosse players.

## Supporting information

S1 File Raw data set. (XLSX)

S2 File Warm up protocol. (DOCX)
