# Supplementary material for: Physiological, perceptual, and technical responses to continuous and intermittent small-sided games in lacrosse players
Source: PLoS One. 2018 Oct 3;13(10):e0203832. doi: 10.1371/journal.pone.0203832 (PMC6169881; doi:10.1371/journal.pone.0203832)
Supplement: S2 File — (DOCX) [file pone.0203832.s002.docx]

Warm up protocol – Small Sided Games Lacrosse

# Lower body tissue quality (Foam Roller) 4 min.

- 1. Calf -> 30 sec. each leg
  2. Hamstring -> 30 sec. each leg
  3. Gluteus -> 30 sec. each leg
  4. Lower back -> 30 sec.
  5. Quadriceps -> 30 sec. each leg

# Activation exercises 4 min.

- 1. Sitting leg rise -> one leg lifting and lowering -> 10 reps each leg
  2. Single leg deadlift with arms stretched out -> 10 reps left leg
  3. Lying lateral leg rise -> upper leg lifting and lowering -> 10 reps each leg
  4. Single leg deadlift with arms stretched out -> 10 reps right leg
  5. Lying lateral leg rise -> lower leg lifting and lowering -> 10 reps each leg
  6. Sitting thoracic spine mobilization -> shorten and extend thoracic spine -> 10 reps
  7. Prone position leg rise -> alternating leg lifting and lowering -> 10 reps

# Correction exercises for ankle and hips 2 min.

Active dorsal flexion of the ankle
Standing position facing a wall (about 20cm distance); alternating knee contact with the wall, with heels on the ground

# Thermogenic and dynamic exercise 10 min.

- 1. Ankle hops forward/backward -> 30 sec.
  2. Duck walk -> 10 meter
  3. Dynamic quadriceps stretches -> walking forward -> 10 meter
  4. Ankle hops left/right -> 30 sec.
  5. Side lunges, alternating leg moving forward -> 10 meter
  6. Diagonal lunges, moving forward -> 10 meter
  7. Ankle hops high and fast -> 30 sec.
  8. Lunges with elbow to the ground rotation -> 10 meter
  9. Dynamic hamstring stretches -> walking forward -> 10 meter
  10. 80% Shuttle run 15+15m -> 30 meter total
  11. Standing scale -> 30 sec. each leg
  12. 80% Shuttle run 15+15m -> 30 meter total
